# *Physaria fendleri FAD3-1* overexpression increases ɑ-linolenic acid content in *Camelina sativa* seeds

**DOI:** 10.1038/s41598-023-34364-9

**Published:** 2023-05-02

**Authors:** Mid-Eum Park, Hyun-A Choi, Hyun Uk Kim

**Affiliations:** 1 Department of Molecular Biology, Sejong University, Seoul, South Korea; 2 Department of Bioindustry and Bioresource Engineering, Sejong University, Seoul, South Korea; 3 Plant Engineering Research Institute, Sejong University, Seoul, South Korea

**Keywords:** Biotechnology, Plant sciences

## Abstract

Camelina (*Camelina sativa*) is an oil crop with a short growing period, resistance to drought and cold, low fertilizer requirements, and can be transformed using floral dipping. Seeds have a high content of polyunsaturated fatty acids, especially ɑ-linolenic acid (ALA), at 32–38%. ALA is an omega-3 fatty acid that is a substrate for eicosapentaenoic acid (EPA) and docosahexaenoic acid (DHA) in the human body. In this study, ALA content was further enhanced by the seed-specific expression of *Physaria fendleri FAD3-1 (PfFAD3-1)* in camelina. The ALA content increased up to 48% in T2 seeds and 50% in T3 seeds. Additionally, size of the seeds increased. The expression of fatty acid metabolism-related genes in *PfFAD3-1 OE* transgenic lines was different from that in the wild type, where the expression of *CsFAD2* decreased and *CsFAD3* increased. In summary, we developed a high omega-3 fatty acid-containing camelina with up to 50% ALA content by introducing *PfFAD3-1*. This line can be used for genetic engineering to obtain EPA and DHA from seeds.

## Introduction

Plant oils are composed of triacylglycerols (TAG), which comprise fatty acids (FAs) that form ester bonds with the glycerol backbone. Plant oils can be used in various ways, such as for food and industrial purposes, depending on their FA composition^1^. Generally, plant oils are mainly composed of five types of FAs: saturated FAs, including palmitic acid (PA; 16:0) and stearic acid (SA; 18:0), and unsaturated FAs, including oleic acid (OA; 18:1), linoleic acid (LA; 18:2), and α-linolenic acid (ALA; 18:3)^2^.

The aim of this study was to enhance the production of omega-3 FAs. The methyl side of FAs is called omega. If there is a double bond at the third carbon of the methyl group, it is called an omega-3 FA, and if it has a double bond at the sixth carbon, it is called an omega-6 FA^3,4^. Omega-3 FAs include ALA, eicosapentaenoic acid (EPA), docosapentaenoic acid (DPA), and docosahexaenoic acid (DHA)^5^. ALA is an essential FA because it is not synthesized in humans^5^. ALA is found in nut and seed oils, while EPA and DHA are mainly found in fish^4,6^. In the human body, ALA is slightly converted into EPA and DHA. The conversion ratio of ALA to EPA is approximately 8%, but the ratio of ALA to DHA is much lower^7,8^. An adequate ratio of omega-3 to omega-6 FAs (1:1 to 1:4) should be maintained in the human body, depending on the disease condition, by consuming foods rich in omega-3 FAs^9^. Fish may be a good source of omega-3 because they contain higher amounts of EPA and DHA^10^. However, it is concerning that omega-3 FAs, such as EPA and DHA, are abundant in fish, but they contain high levels of mercury. In addition, vegetarians do not eat fish^6^. Therefore, plant-derived omega-3 FAs are a good alternative to fish-derived omega-3 FAs.

Fatty acid desaturase 3 (*FAD3*) was identified to synthesize ALA in Arabidopsis (*Arabidopsis thaliana*). FAD3 synthesizes the ALA by desaturation of 18:2, located at *sn-2* of phosphatidylcholine (PC) in the endoplasmic reticulum (ER) membrane^11,12^. *FAD3* contains three histidine boxes with catalytic activities that are conserved in most plants^13^. In addition, *FAD3* contains several transmembrane domains that differ between plant species, and functional studies of the *FAD3* gene have been conducted in various plants, including Arabidopsis^14–24^. Various *FAD*3s isolated from different plant species were overexpressed in several other plant species to study their effects on FA content and other traits. Overexpression of *Plukenetia volubilis FAD3* in tobacco seeds decreased 18:2 and increased 18:3 when compared to that in the wild type (WT)^20^. Overexpression of Perilla *FAD3* in Arabidopsis significantly increased 18:3 content^21^. Overexpression of *Brassica napus FAD3* in tobacco not only increased 18:3 content but also improved drought resistance^23^. Similarly, the overexpression of *Arachis hypogaea* L *FAD3* in Arabidopsis led to an increase in 18:3 and salt resistance^17^. However, overexpression of camelina *FAD3* in Arabidopsis increased the 18:3 content but also resulted in wrinkled seeds and reduced oil content and seed weight^24^.

*Physaria fendleri FAD3-1* (*PfFAD3-1*) was used to increase ALA levels in camelina seeds. *PfFAD3-1* was chosen because soybean has a low 18:3 content; however, the expression of *PfFAD3-1* in soybean significantly increased the 18:3 content by up to 52.4%^14,25,26^. *P. fendleri* is a plant species belonging to the Brassicaceae family, with oil containing 60% lesquerolic acid, which is a hydroxy fatty acid^27,28^. PfFAD3-1 and PfFAD3-2 are two FAD3s found in *P. fendleri*, both of which are ER membrane-bound proteins with three histidine boxes and four transmembrane domains^14,25^. Recently, *PfFAD3-1* and *PfFAD3-2* were expressed in the Arabidopsis *fad3-2* mutants. PfFAD3-1 restored the 18:3 content by up to 29.9% when expressed in the *fad3-2* mutant, which has a low ALA content, whereas PfFAD3-2 has little desaturase activity^14^.

Camelina has become an increasingly important research tool for studying biofuels and other biotechnological applications of oil crops^29–31^. It is an allohexaploid (2n = 6x = 40) plant belonging to the Brassicaceae family that is grown primarily in Europe and North America^32,33^. Camelina has a short life cycle (85–100 days), low fertilizer requirements, and resistance to cold and drought^33^. It can be easily transformed using the floral dipping method^34^. Polyunsaturated FAs constitute approximately 50% of camelina seeds; particularly, 32–38% of 18:3 FA. The 18:1 FA and 20:1 FA contents are 12–18% and 12–17%, respectively^33^.

**Figure 1:**
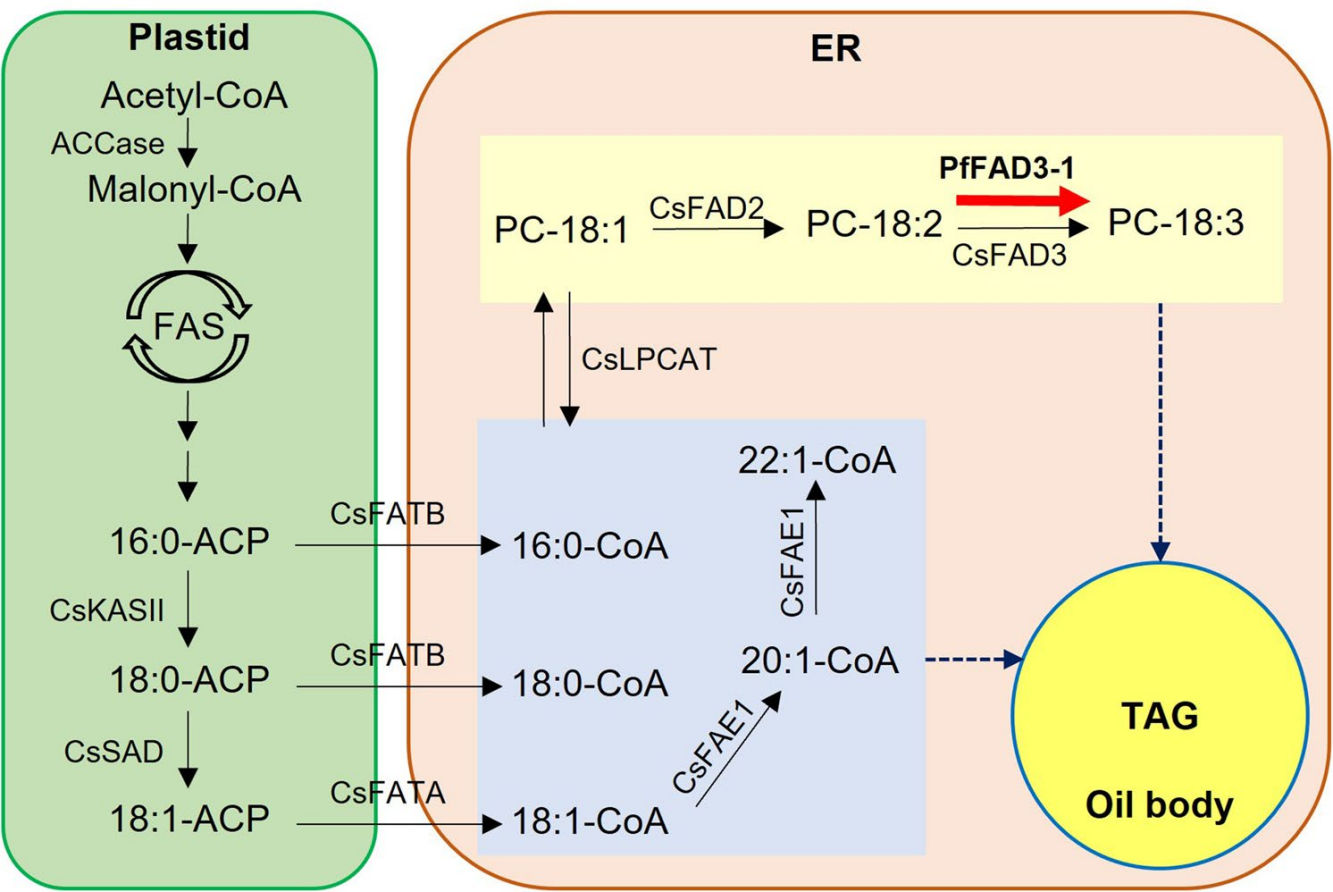
Schematic diagram of fatty acid synthesis pathway in *Camelina sativa* seeds. Fatty acid (FA) synthesis in the plastids. Acetyl-CoA is converted to malonyl-CoA by ACCase and then elongated by FAS, KASIII, and KASI. KASII elongates palmitic acid (16:0) to stearic acid (18:0). SAD catalyzes the desaturation of stearic acid into oleic acid (18:1). FAs are transported to plastids by FATA and FATB. Linoleic acid (18:2) and α-linolenic acid (18:3) are synthesized from oleic acid in the PC membrane of the ER by FAD2 and FAD3 enzymes. In addition, oleic acid can be elongated into eicosenoic acid (20:1) and erucic acid (22:1) by FAE1. Black arrows indicate FA flow, and red arrows show the pathway for enhancing linolenic acid by *PfFAD3-1* expression. The blue dotted arrow indicates the process by which various FAs transfer to TAG by several enzymes. ACP, acyl carrier protein; CoA, coenzyme A; ER, Endoplasmic reticulum; FAD2, fatty acid desaturase 2; FAD3, fatty acid desaturase 3; FAE1, fatty acid elongase 1; FAS, fatty acid synthase; FATA, fatty acyl-ACP thioesterase A; FATB, fatty acyl-ACP thioesterase B; KASII, β-ketoacyl-acyl carrier protein synthase II; LPCAT, acyl-CoA:lysophosphatidylcholine acyltransferase; SAD, stearoyl-ACP desaturase; TAG, triacylglycerol.

FA biosynthesis is initiated by the conversion of acetyl-coenzyme A (CoA) to malonyl-CoA in the plastids by acetyl-CoA carboxylase (ACCase)^35^. Malonyl-CoA is then converted into malonyl-ACP by malonyl-CoA: acyl carrier protein (ACP) transacylase^36^. Malonyl-ACP is converted to 4:0-ACP by FA synthase (FAS) and ketoacyl-acyl carrier protein synthase (KAS) III and is synthesized as 16:0-ACP by KAS I and FAS enzymes^37^. Thereafter, it is synthesized as 18:0-ACP by FAS and KASII. Stearoyl-ACP desaturase (SAD) generates 18:1-ACP from 18:0-ACP^37^. Fatty acyl-ACP thioesterase B (FATB) specifically cleaves 16:0-ACP and 18:0-ACP, which are saturated FAs, while 18:1-ACP is specifically cleaved by fatty acyl-ACP thioesterase A (FATA). The three free FAs produced by FATA and FATB are exported from the plastid to the cytosol^38^. Free FAs (16:0, 18:0, and 18:1) combine with CoA in the cytosol to form an acyl-CoA pool. 18:1-CoA is synthesized into 20:1-CoA and 22:1-CoA in the cytosol by fatty acid elongase 1 (FAE1)^39^. The desaturation of 18:1 to 18:2 and 18:3 involves a sequential reaction of *FAD2* and *FAD3*, which target 18:1 at the *sn-2* position of PC. The 18:1, 18:2, and 18:3 FAs in PC are released into the acyl-CoA pool via the reverse reaction of lysophosphatidylcholine acyltransferase (LPCAT)^40^. Various FAs in the acyl-CoA pool are transferred to glycerol to form TAG using glycerol-3-phosphate acyltransferase (GPAT), lysophospholipid acyltransferase (LPAT), and diacylglycerol acyltransferase (DGAT)^35^. In this study, *PfFAD3-1* was introduced into camelina to increase ALA content in seeds (Fig. 1).

## Results

### FA composition analysis in seeds across T1 to T3 generations of *PfFAD3-1* overexpressed camelina

**Figure 2:**
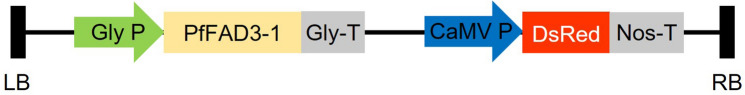
A vector map of *Physaria fendleri FAD3-1* (*PfFAD3-1*) for seed-specific expression. CaMV P, cauliflower mosaic virus promoter; DsRed, red fluorescent protein; Gly P, glycinin promoter; Gly-T, glycinin terminator; LB, left border; RB, right border; Nos-T, nopaline synthase terminator.

**Figure 3:**
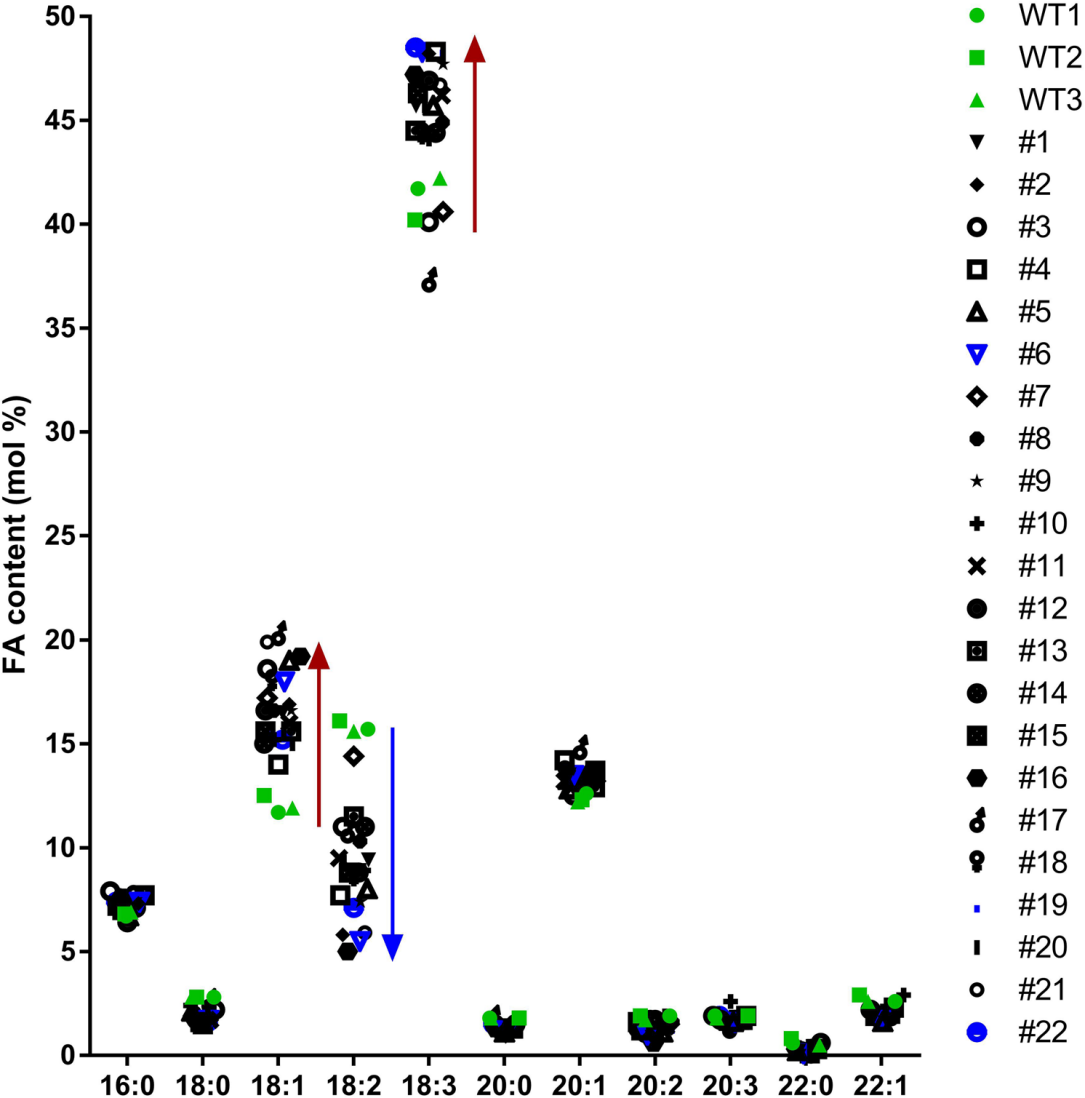
Fatty acid composition of *Camelina sativa* cv. Suneson and T1 transgenic lines expressing *PfFAD3-1* in T2 seeds. FAME was analyzed in the T2 generation seeds of T1 transgenic plants and compared with the WT. Green points indicate the WT. Black points indicate transgenic plants, and *PfFAD3-1 OE* #6, #19, and #22 lines are marked in blue. *PfFAD3-1 OE* #6, #19, and #22 lines were selected for T2 generation.

*PfFAD3-1* was overexpressed in camelina to enhance ALA production in seeds. The pBinGlyRed3 vector was used for seed-specific expression^41^. This vector contains a glycinin promoter, a seed-specific promoter, and DsRed (a red fluorescent protein) as a selection marker. *PfFAD3-1* complementary DNA (cDNA) was cloned into pBinGlyRed3 vector (Fig. 2). The FA composition of the T2 seeds of 22 plants was analyzed and compared with that of the WT to observe changes in FA content in *PfFAD3-1 OE* lines (Fig. 3, Table S1). Transgenic plants are denoted as *PfFAD3-1 OE* numbers in the following description. In most transgenic plants, 18:3 content was increased compared to that in the WT, leading to a decrease in 18:2 content, which is a substrate of *FAD3*. The average 18:3 content in the WT was 41.4% and 45.5% in the transformant, indicating an increase of approximately 4%. Furthermore, the average 18:2 content in the WT was 15.8% and 8.7% in the transformant, showing a reduction of approximately 7%. The average 18:1 content was 12% in the WT but increased to an average of 16.9% in the transformant. The contents of other FAs did not significantly differ between the WT and transgenic plants (Fig. 3).

The T1 generation was not a homozygous line; therefore, only *PfFAD3-1 OE* #6, #19, and #22 lines, which showed the highest 18:3 contents of 48.3%, 48.4%, and 48.5%, respectively, among the 22 transformants, were selected and progressed to the T2 generation. The FA composition was analyzed using gas chromatography (GC) in the T3 seed (Table S2). The average 18:3 content in the WT was 35.7%, whereas that in the *PfFAD3-1 OE* #6, #19, and #22 lines was 46.1%–49.8%. The average 18:2 content was 16.3% in the WT, whereas that in the *PfFAD3-1 OE* #6, #19, and #22 lines was 2.9%–4.1%, showing a decrease of more than 12%.

**Figure 4:**
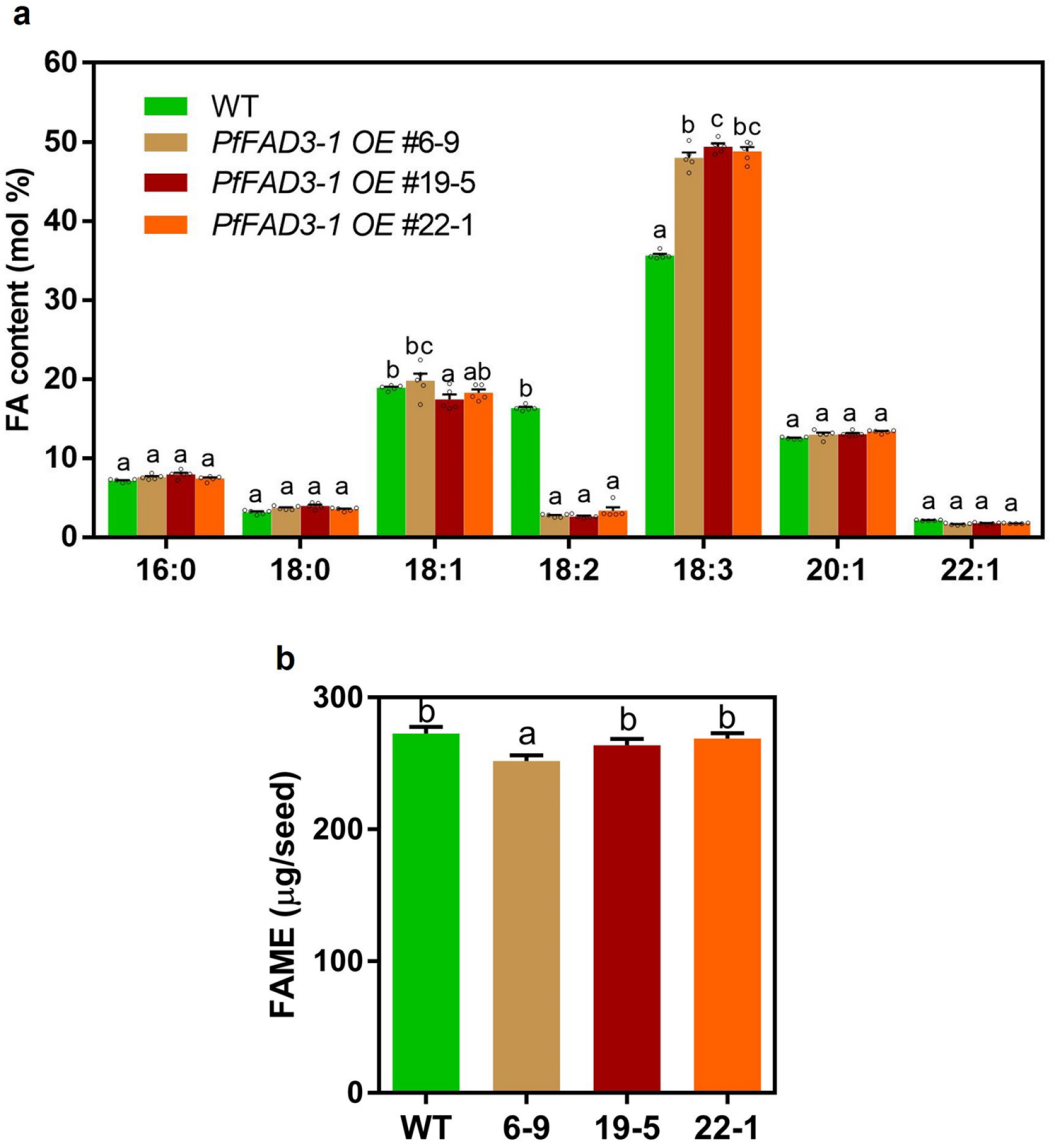
Fatty acid composition and oil content in T3 seeds of T2 generation transgenic lines. (**a**) FA composition of three transgenic (*PfFAD3-1 OE* #6-9, #19-5, #22-1) and WT plants. (**b**) Total FAME (µg/seed) of transgenic and WT plants. The results were analyzed as five replicates (*n* = 5). Error bars represent standard error of the mean (SEM). Statistical significance is indicated by different letters using one-way ANOVA and Tukey’s multiple comparison test (*p < 0.05).

FA analysis was performed five times to determine the FA composition and oil content of the T3 generation lines *PfFAD3-1 OE* #6-9, #19-5, and #22-1 (Table S3). The average 18:3 content was 48%, 49.4%, and 48.8%, respectively, which was significantly higher than that of the WT (35.7%) (Fig. 4a). The contents of saturated FAs 16:0 and 18:0 in the WT were 7.1% and 3.2%, respectively, and those in the *PfFAD3-1 OE* #6-9, #19-5, and #22-1 lines were 7.4–7.9% and 3.5–4.0%, respectively, showing no significant differences from those in the WT (Fig. 4a). The 18:2 content in the WT was 16.3%, whereas in *PfFAD3-1 OE* #6-9, #19-5, and #22-1 it was 2.6–3.4% (Fig. 4a). Furthermore, the 20:1 and 22:1 content in the WT were 12.5% and 2.1%, respectively, and those in *PfFAD3-1 OE* #6-9, #19-5, and #22-1 were 13.0–13.3% and 1.6–1.7%, showing no significant differences compared to that in the WT. In contrast, the 18:1 content in the WT was 18.9%, but increased slightly to 19.8% in the *PfFAD3-1 OE* # 6-9 lines and decreased in *PfFAD3-1 OE* #19-5 (17.4%) and 22-1 (18.2%) lines (Fig. 4a, Table S3).

The total oil content of the seeds was measured as the total fatty acid methyl ester (FAME). The FAME content of WT was 272.5 μg/seed, and that of *PfFAD3-1 OE* #6-9, #19-5, and #22-1 lines was 251.8, 263.8, and 268.9 μg/seed, respectively (Fig. 4b). In the *PfFAD3-1 OE* #19-5 and #22-1 lines, the total FAME (μg/seed) content was similar to that in the WT; however, the total FAME in the *PfFAD3-1 OE* #6-9 line decreased by 8% compared to that in the WT (Fig. 4b).

In summary, the results of the FA analysis up to the T3 generation revealed that seed-specific overexpression of *PfFAD3-1* enhanced 18:3 content in seeds and decreased 18:2 content. Moreover, the effect of *PfFAD3-1* was confirmed to be maintained until the T3 generation.

### Seed phenotype of *PfFAD3-1 OE* lines in T3 generation

**Figure 5:**
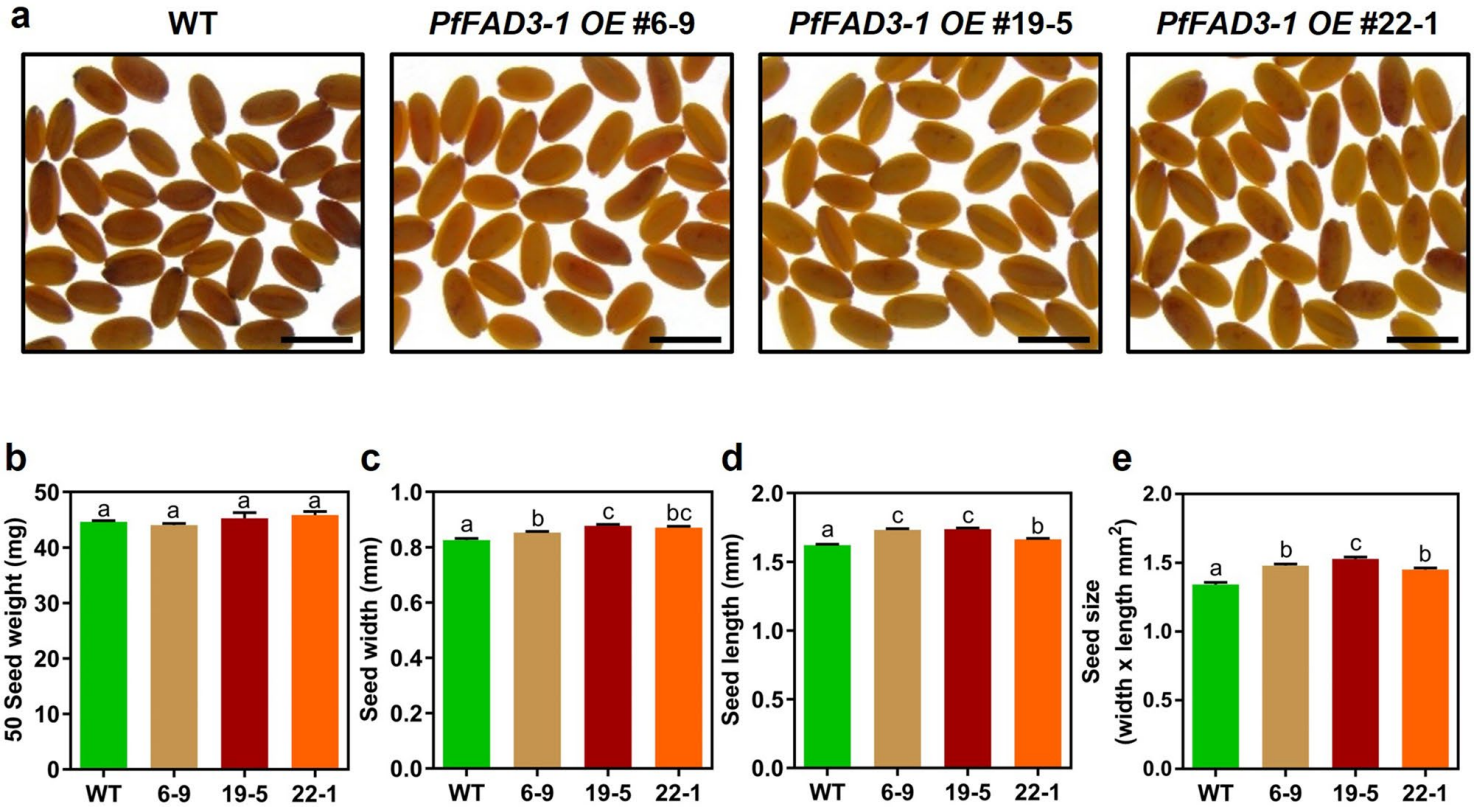
Seed phenotype of transgenic and wild type plants in the T3 generation. (**a**) Images of seeds of transgenic (*PfFAD3-1 OE* #6-9, #19-5, #22-1) and WT plants. (**b**) Seed weight. (**c**) Seed width. (**d**) Seed length. (**e**) Seed size. The results were analyzed in triplicates (*n* = 30). Scale bar = 2 mm. Error bars represent SEM. Statistical significance is indicated by different letters using one-way ANOVA and Tukey’s multiple comparison test (*p < 0.05).

Seed weight and size were analyzed to investigate the effect of overexpressing *PfFAD3-1* on camelina seeds. Seed width, length, and size were measured in triplicate for 50 seeds per line (Fig. 5a). There was no significant difference in seed weight between the WT and *PfFAD3-1* OE lines (Fig. 5b). However, the seed width and length of all three transgenic lines were significantly greater than those of the WT (Fig. 5c,d). The average seed width of the WT was 0.83 mm, whereas that of *PfFAD3-1* OE #6-9, #19-5, and #22-1 lines was 0.85, 0.88, and 0.87 mm, respectively (Fig. 5c). The average seed length of the WT was 1.62 mm, whereas that of *PfFAD3-1* OE #6-9, #19-5, and #22-1 lines was 1.73, 1.74, and 1.66 mm, respectively (Fig. 5d). Seed size was calculated by multiplying the width and length, which showed an increase in the transgenic lines compared with that in the WT (Fig. 5e).

### Analysis of FA composition and FA synthesis-related gene expression in the seed development stage of *PfFAD3-1 OE* lines

**Figure 6:**
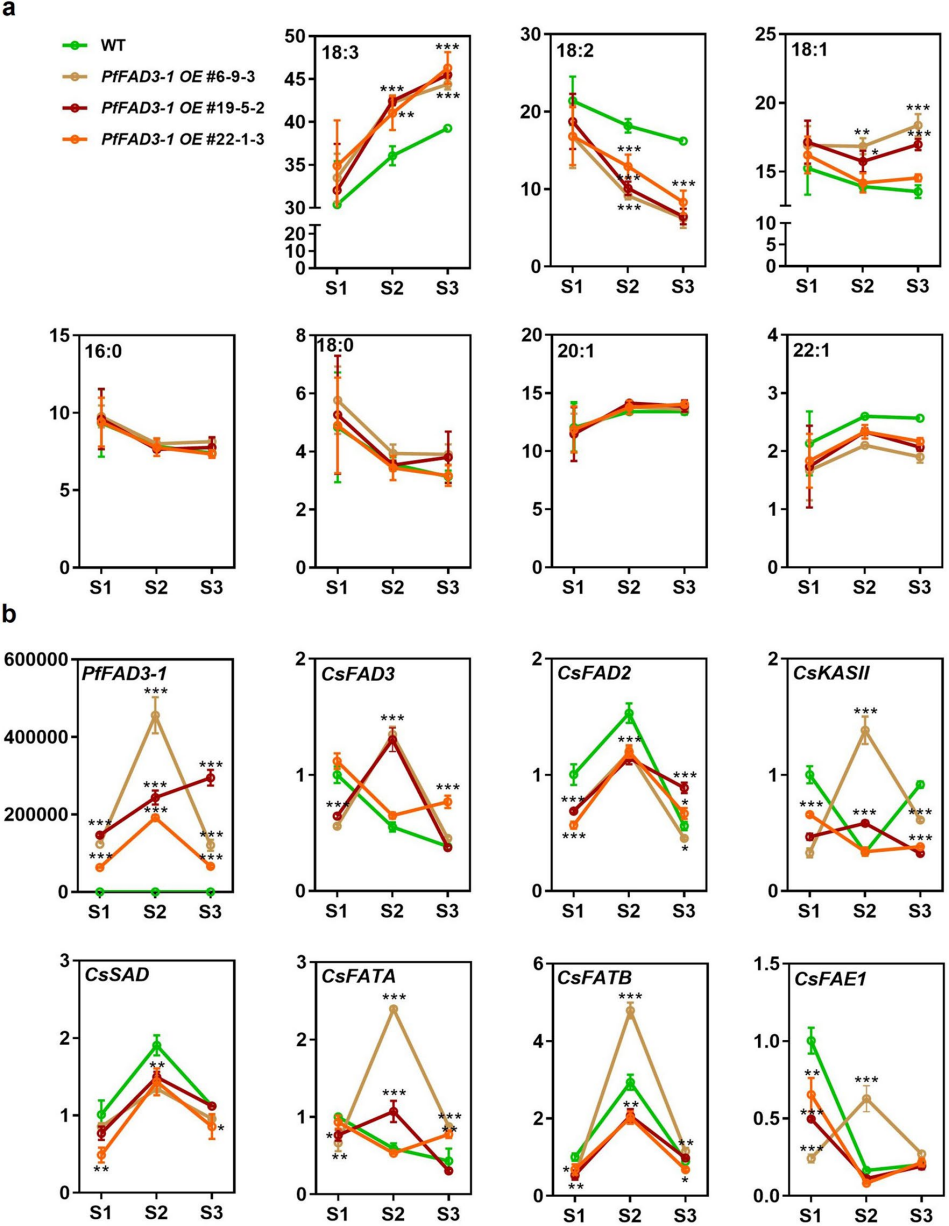
Fatty acid composition and gene expression in the stage of T4 generation developing seeds. (**a**) FA composition in transgenic and WT plants. (**b**) Gene expression patterns in the developing stage of transgenic lines and WT. S1, S2, and S3 were developing seeds harvested 30, 40, and 50 days after flowering, respectively. Error bars represent SD. Statistical analysis was performed using one-way ANOVA with Tukey’s multiple comparison test (*p < 0.05, **p < 0.01, ***p < 0.001).

Seven genes (*KASIII, SAD, FATA,* and *FATB*, which are involved in FA biosynthesis; *FAD2* and *FAD3,* which are FA desaturase genes; and *FAE1*, which is an elongase gene) were selected to investigate whether the introduction of *PfFAD3-1* affected the expression of endogenous FA synthesis genes in camelina (Fig. 6). Seed development was divided into three stages (S1, S2, and S3), and FA composition and expression of FA biosynthesis genes in the seeds were analyzed for the *PfFAD3-1 OE* #6-9-3, #19-5-2, and #22-1-3 lines of the T4 generation (Fig. 6a,b).

In the WT and three *PfFAD3-1 OE* lines, 16:0 FA and 18:0 FA continued to decrease from S1 to S3, showing no significant differences. The 18:1 content decreased from S1 to S2 and increased in the S3 stage, and the three transgenic plants showed higher content than the WT plants at all stages. The opposite trend was observed between the 18:2 and 18:3 contents; the 18:2 content decreased from S1 to S3, whereas the 18:3 content increased. The decrease and increase in 18:2 and 18:3 content, respectively, in the transformants gradually differed from those in the WT as seed development progressed. The 20:1 and 22:1 contents tended to increase from the S1 to S2 stages, and then slightly decreased at the S3 stage. However, there was no significant difference between the WT and transformants for either 20:1 FA or 22:1 FA (Fig. 6a).

The expression of FA biosynthesis genes in camelina was analyzed in WT and *PfFAD3-1 OE* plants at each stage of seed development (Fig. 6b). The expression of *PfFAD3-1* was not detected in the developing seeds of the WT plants; however, *PfFAD3-1* was highly expressed in the developing seeds of all three transgenic lines. *CsFAD3*, which has the same function as *PfFAD3-1*, showed different expression patterns between the WT and transgenic plants. At the S2 and S3 stages, the transgenic plants showed a 2.4-fold upregulation of *CsFAD3* compared to the that in the WT. The expression patterns of *CsSAD*, *CsFATB*, and *CsFAD2* showed the same tendencies in the WT and the transformants. However, the expression levels of *CsSAD* and *CsFAD2* were 0.7-fold lower in transgenic plants than in WT plants at S2 stage. During the S2 stage, *CsFATB* expression was elevated 1.6-fold in the *PfFAD3-1 OE #6-9-3* line compared to the WT; however, it showed a 0.4-fold decrease in the *PfFAD3-1 OE* #19-5-2 and #22-1-3 lines compared to that in the WT. *CsKASII, CsFATA*, and *CsFAE1* showed different expression patterns in the WT and transgenic plants (Fig. 6b). In summary, 18:3 content increased and 18:2 content decreased in the developing seeds of transgenic plants compared to that in WT plants. This was due to the introduction of *PfFAD3-1,* and the expression of exogenous *PfFAD3-1* induced the upregulation of *CsFAD3* and downregulation of *CsFAD2*.

## Discussion

In this study, *PfFAD3-1*, which converts 18:2 FA to 18:3 FA, was transformed into camelina (Fig. 1). Consequently, the ALA (18:3) content increased by up to 48% in the T2 seeds and up to 50% in the T3 seeds of the transgenic camelina plants (Figs. 3, 4). The *PfFAD3-1* OE lines showed an average increase of 12.3–13.7% in 18:3 content and a decrease of 13.0–13.7% in 18:2 content (Table S3). This similarity in the increase of 18:3 and decrease of 18:2 suggests that the conversion of 18:2 FA to 18:3 FA in camelina by *PfFAD3-1* is both specific and efficient. In a previous study, when *PfFAD3-1* was transformed into soybeans under the control of the seed-specific promoter, β-conglycinin, the ALA content increased by up to 52% in T1 seeds and up to 42% in T2 seeds^26^. In soybean, *PfFAD3-1* expression increased ALA content under the control of the phaseolin promoter and 35S promoter; however, the increase in ALA content was the greatest under the control of β-conglycinin^26^. *PfFAD3-1* expression in soybeans did not result in significantly different oil content from that of the WT; however, it increased both seed size and weight^26^. Similarly, in this study, the seed oil content of *PfFAD3-1 OE* #6-9 line was slightly lower than that of the WT, whereas that of *PfFAD3-1 OE* #19-5 and #22-1 lines was similar to that of the WT (Fig. 4b). In the *PfFAD3-1 OE* #6, #19, and #22 lines, some plants in the next generation exhibited lower ALA content than those in the previous generation, as indicated in Table S2. This may be due to the presence of non-homozygous plants or variation in transgene expression among individuals. Nonetheless, the average ALA content of the progeny of these lines ranged from 46.1 to 49.8%, which was similar or slightly higher than that of the previous generation (Table S2). These findings suggest that the transmission and expression of the *PfFAD3-1* transgene is stable and heritable in subsequent generations. Seed weight did not differ between the WT and the three transgenic lines. However, seed width, length, and size increased compared with those of the WT (Fig. 5b–e). This showed a seed phenotype similar to that observed when *PfFAD3-1* was expressed in soybean^26^. Hence, the seed-specific expression of *PfFAD3-1* in camelina did not significantly affect seed weight but increased seed size. Since seed size increased without a significant effect on seed oil content, further research is required to determine whether ALA content affects seed size.

Camelina seed oil can be used for various purposes such as baking, frying, and salad dressings^42^. The camelina developed in this study is beneficial for health because it contains up to 10% or more omega-3 FA compared to that in the WT. Camelina seeds contain eicosenoic acid (20:1) and erucic acid (22:1) at 12–17% and 2–3%, respectively^33^. The 20:1 and 22:1 FAs were synthesized from 18:1 by FAE1^43^. Camelina was identified as having three copies of *FAE1* genes^39^. When three copies of *FAE1* were knocked out in camelina using the CRISPR/Cas9 technology, 20:1 and 22:1 synthesis was blocked, thus increasing the 18:1 content and continuously increasing the ALA content up to 50%^44^. In this study, *PfFAD3-1* expression increased 18:3 content by up to 50% (Fig. 4). Therefore, knocking out *CsFAE1* using CRISPR/Cas9 in the *PfFAD3-1* overexpression line will further increase the ALA content by over 50%. In addition, under *PfFAD3-1* expression, the 18:2 content was 2.6–3.4%, indicating that *PfFAD3-1* sufficiently synthesized 18:3 using 18:2 as a substrate. Therefore, if the *FAD2* gene is additionally overexpressed in the *PfFAD3-1* overexpression line, the flux from 18:1 to 18:2 would increase; thus, PfFAD3-1 could use 18:2 to further enhance ALA content.

Since humans cannot synthesize ALA, it must be obtained through dietary sources, such as rapeseed or walnuts, which have high ALA content^45^. This study successfully developed a high-ALA camelina that could be used as a source of plant oil for ALA intake. From a clinical standpoint, increasing the ALA intake is important because it has been shown to reduce the risk of incident stroke^46^. While there are suggestions that ALA may have positive effects on cardiovascular health, the results are conflicting, indicating the need for further research in this area^47^. In addition to its direct health benefits, ALA can be converted into EPA and DHA in the human body, which also have beneficial effects. However, the efficiency of this conversion process is typically low^8^. To address this, research has been conducted to develop crops that can produce EPA and DHA directly from ALA through the expression of the desaturase and elongase enzymes involved in the synthesis of these FAs^48–50^. Recently, a transgenic camelina strain was developed using multigene metabolic engineering to produce 12% DHA in seeds^49^. In addition, this line was crossed with *CsFAE1* knockout camelina containing high ALA content in seeds, further increasing omega-3 FA content (ETA, EPA, DPA, and DHA) from 27 to 33%^51^. Therefore, the high-ALA camelina developed in this study can be used to produce omega-3 FAs such as EPA and DHA.

## Materials and methods

### Vector construction of *PfFAD3-1* gene

cDNA was synthesized using mRNA from developing seeds of *P. fendleri* using a PrimeScript 1st strand cDNA synthesis kit (Takara, Japan). *P. fendleri* seed WCL-LY1 (PI596362) was provide by the U.S. National Plant Germplasm System (NPGS). The synthesized cDNA was used as a template, and polymerase chain reaction (PCR) was performed using primers targeting the *PfFAD3-1* open reading frame (Table S4). The *PfFAD3-1* sequence was obtained from a previous study^14^. The PCR product was eluted using a PCR purification kit (Cosmogenetech, Korea) and cloned into the pGEMT-Easy vector (Promega, USA). Thereafter, we checked whether there was a PCR error using Sanger sequencing and proceeded to the next step. There is an *Eco*RI enzyme site on both sides of the pGEMT-Easy vector and a multi-cloning site, including the *Eco*RI enzyme site, in the pBinGlyRed3 vector equipped with a seed-specific glycine promoter. Therefore, it was cut with *Eco*RI enzyme, and the PCR product was ligated into the pBinGlyRed3 vector.

### Selection of *PfFAD3-1* transformed camelina

*Camelina sativa* cv. Suneson was used for all the experiments in this study. Camelina seeds were provided by Dr. Edgar Cahoon (University of Nebraska-Lincoln, Lincoln, Nebraska, USA). Camelina seeds were germinated on filter paper containing water in a culture chamber at 16 °C under a 16 h light/8 h dark photoperiod. After one week, the seeds from which the cotyledons emerged were transplanted into the soil. Eighteen plants were grown in soil, and transformation was performed using the floral dipping method^34^. The *Agrobacterium* strain GV3101 was used. *Agrobacterium* cells (500 μl) and kanamycin antibiotics (500 μl) were added to 500 ml LB medium and incubated overnight at 28 °C until an optical density value of 1 was obtained. This solution was centrifuged at 3000 × *g* for 10 min and resuspended in 500 ml solution containing 1% sucrose and 0.05% Silwet L-77. The camelina flowers were then immersed in this solution for 1 min. After floral dipping, plants were placed in a growth chamber for 24 h in the dark. This procedure was performed three times at intervals of 5 d. After harvesting the transformed plants, 22 fluorescent seeds were selected using a green flashlight and a red filter. After germination, these 22 T1 lines and the WT were grown in growth chamber at 20 °C under a 16 h light/8 h dark photoperiod to obtain the next generation. All procedures, including the collection of plant materials and experimental research, were conducted in accordance with institutional, national, and international guidelines.

### Analysis of gene expression in *PfFAD3-1 OE* lines

Reverse transcription real-time PCR (RT-qPCR) was performed on RNA extracted using the method described in the reference^52^. Developing seeds were taken at the beginning of the desiccation phase (30, 40, and 50 days after flowering) based on reference^53^. After RNA isolation, 2 μg cDNA was synthesized using a cDNA synthesis kit (Takara, Japan). RT-qPCR analysis was performed using the synthesized cDNA as a template and SYBR Green Master Mix (TOYOBO, Japan) in a StepOnePlus Real-Time PCR System (Thermo Fisher Scientific, USA). Camelina *ACTIN2* was used as an endogenous gene control, and primers capable of targeting all three copies of the genes were designed and used in this study (Table S4).

### Fatty acid analysis

Seven camelina seeds were subjected to GC. Seeds were placed 500 μl of toluene and 1 ml of 5% H_2_SO_4_ including pentadecanoic acid (15:0) standard (100 μg/ml) and allowed to react for 2 h in an 85 °C water bath. When the reaction was complete, 1 ml of 0.9% NaCl and 1 ml of hexane were added, mixed thoroughly, and centrifuged at 330 × *g* for 2 min. The supernatant was transferred to a 6 ml tube. The procedure of adding hexane and centrifuging was repeated three times. A total of 3 ml of the supernatant was purged with nitrogen gas, dissolved in 200 µl hexane, and transferred to a GC vial. A DB-23 column (30 m × 0.25 mm, 0.25 μm film, Agilent, USA) was used, and analysis was performed using a GC-2030 instrument (Shimadzu, Japan). The GC oven temperature was increased from 190 to 230 °C at a rate of 5 °C per min.

### Analysis of seed phenotype

Using the WT as a control, seed weight, length, width, and size were measured for the transgenic line. Seeds from each line were analyzed in triplicate to determine the seed phenotype. Seed photographs were captured using an optical microscope (NIKON, Japan). Seed weight was measured using an electronic scale (OHAUS, USA). Seed length and width were measured using ImageJ software, and seed size was calculated by multiplying the seed length and width.

### Statistical analysis for data

Statistical analyses were performed using one-way analysis of variance (ANOVA) and Tukey’s multiple comparison test using GraphPad Prism to determine the significance of the differences. Different letters indicate significant changes compared to the WT plants in the FA and seed phenotype analyses. Significant differences in FA composition and gene expression patterns at different seed developmental stages are indicated by *p < 0.05, **p < 0.01, and ***p < 0.001.

## Supplementary Information

Supplementary Tables.

## Data Availability

All data generated or analyzed in this study are included in this published article (and its supplementary information files).
